# Novel 60 GHz Band Spatial Synthetic Exposure Setup to Investigate Biological Effects of 5G and Beyond Wireless Systems on Human Body

**DOI:** 10.3389/fpubh.2021.777712

**Published:** 2021-12-06

**Authors:** Takashi Hikage, Ryunosuke Ozaki, Tatsuya Ishitake, Hiroshi Masuda

**Affiliations:** ^1^Faculty of Information Science and Technology, Hokkaido University, Hokkaido, Japan; ^2^Department of Environmental Medicine, Kurume University School of Medicine, Fukuoka, Japan

**Keywords:** human exposure, millimeter-waves, biological effects, thermal perception, safety guideline

## Abstract

The global spread of 5th generation (5G) wireless systems causes some concern about health effects of millimeter waves (MMW). To investigate biological effects of local exposure to 5G-MMW on human body, a novel 60 GHz band exposure setup was developed, and its performance was validated. A spatial synthetic beam-type exposure setup using two dielectric lens antennas was proposed to achieve high intensity 60 GHz irradiation to the target area of human skin. Variety distributions and intensities of electromagnetic fields at the exposed area, which is modified by incident angles of the combined beams, were simulated using finite-difference time-domain methods. The exposure performance we estimated was verified by temperature elevations of surface in a physical arm-shaped silicone phantom during the MMW exposure. The interference fringes generated in the exposed area due to the combined two-directional beam radiations were observed both in the simulation and in the phantom experiment but eliminated by applying an orthogonalizing polarized feeding structure. Under these exposure conditions, the local temperature changes, which could evoke warmth sensations, were obtained at the target area of the human forearm skin, which means the achievement of exposure performance we intended.

## Introduction

The use of millimeter waves (MMW) such as 5th generation (5G) wireless systems and WiGig (IEEE 802.11ad) is now widely expanding. But this causes some new concerns about possible adverse health effects on the human body. To provide protection against known adverse health effects, the International Commission on Non-Ionizing Radiation Protection (ICNIRP) established guidelines (1), which specify the basic restriction values in terms of specific absorption rates (SAR, W/kg) or absorbed power density (W/m^2^) for occupational or general public. For example, the restriction values in occupational and general public exposures for the local exposure (6–300 GHz) including the MMW band are 100–20 W/m^2^, respectively. These restriction values are based on evidence of microwave-induced thermal effects and dosimetric findings of thermal changes (2–5) and also include the safety factor considered with the individual differences and age.

However, novel biological evidence is now required for the validation or revision of the guidelines. In particular, it is imperative to accumulate the evidence for 5G-MMW exposure because of its rapid expansion. In addition, 5G-MMW is known to be absorbed mainly into the body surface, but not penetrate into deep tissues (6). Thus, direct effects of the exposure on skin tissue or indirect effects on other physiological functions *via* the skin should be focused on. Several research groups have been exploring the biological effects of exposure to 5G-MMW so far. But these are mainly examined for eyes (7, 8) and cultured cells (9, 10). In contrast, there is little information about the effects on human body surface (6) that include the regulations of skin blood flow or sweating. Moreover, the physiological responses to the differences in intensity or signal modulation of the exposure are neither clear. Therefore, these physiological data under the 5G-MMW exposure could be helpful for the reevaluation of the safety guidelines.

There have been several technical difficulties to advance the studies on human exposure to 5G-MMW. For example, one is about the size of amplifier. A wide range of exposure intensity including high level is needed to obtain a dose-dependent response of physiological parameters. But the high-power amplifier that can generate the modulated MMW is not so very small that its installation with other experimental equipment will be trouble for a limited place of general laboratories. Another difficulty is to provide a sufficient space between the wave source and the exposure target. Some physiological parameters such as skin temperature and blood flow are observed as two-dimensional images using infrared cameras (11). In this case, a sufficient distance from the exposure systems to the target is required not to interrupt a field of vision for the camera. However, this brings a contradictory problem because the longer distance to the target attenuates of the exposure intensity.

The aim of this study was to develop exposure systems to investigate the biological effects of local 5G-MMW exposure on human skin and to validate its performance. To overcome the difficulties mentioned above, we devised a novel spatial synthetic exposure setup for 60 GHz band, which is a candidate frequency band in the 5G and beyond wireless systems. The main components of the setup were two dielectric lenses of antennas, which can irradiate focused beam on required exposure area, and parallel amplifier circuits. Using a physical phantom and human volunteers, we experimentally confirmed whether the setup provided the localized exposure without interference fringes and sufficient intensity for applying to the human study.

## Development of 60 GHz Band Spatial Synthetic Exposure Setup

### Performance Requirement for the Exposure Setup

To investigate biological effects of the local MMW exposure on human body, we have a plan to measure several physiological parameters simultaneously during the exposure. For example, skin temperature and warmth sensation are must for the evaluation to be compared with the previous study (6). Heat pain, skin blood flow, sweating, and heart rate are also the possible candidates to find the effects. In particular, the latter parameters may need higher temperature elevation than the thermal threshold in human skin (12). Moreover, changes in skin temperature and skin blood flow are most likely to be informative if they are observed as two-dimensional images by cameras.

**Figure 1:**
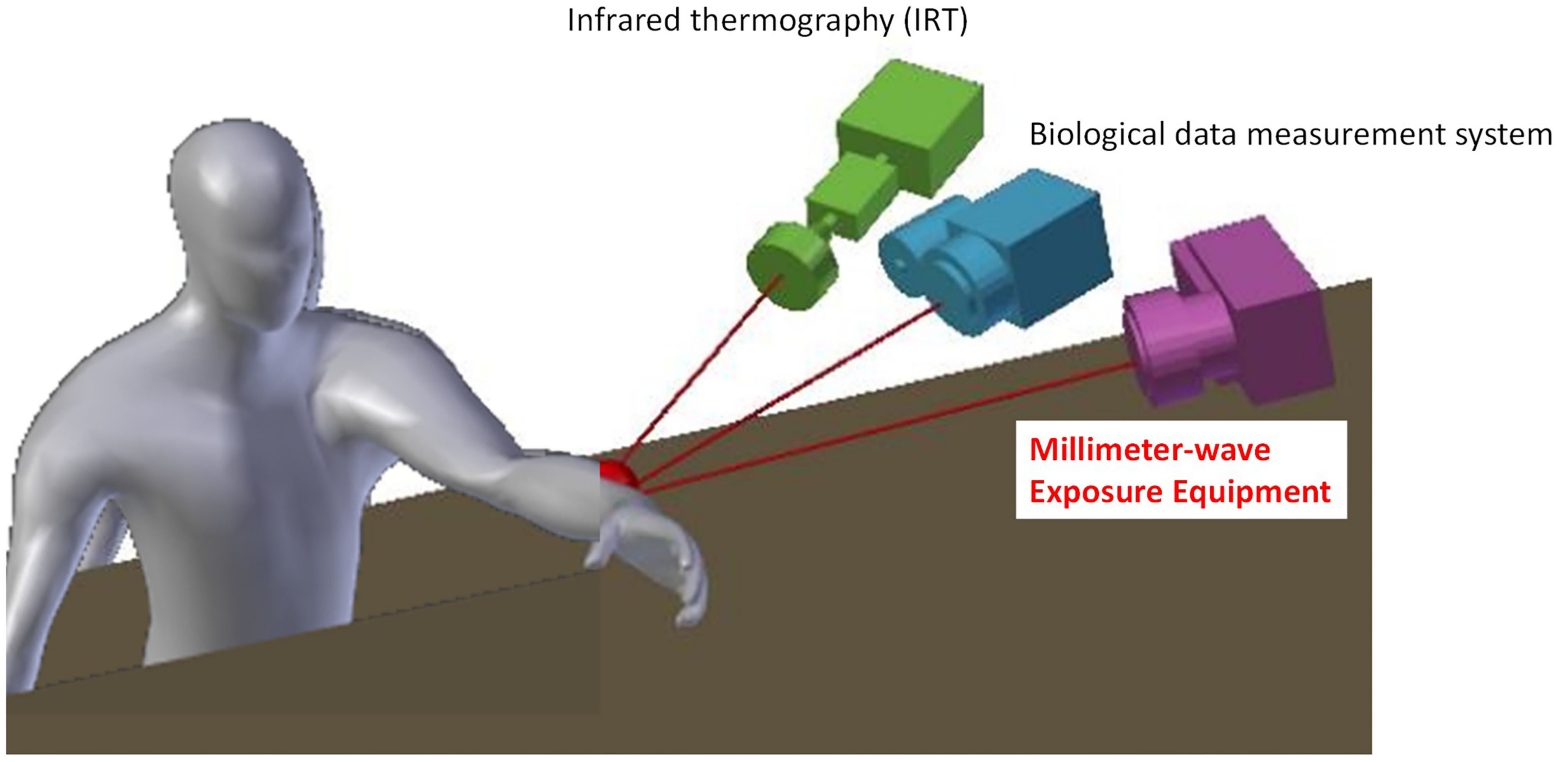
Overview of experimental design to investigate biological effects of 60 GHz MMW exposure on human body.

Then, real-time measurement systems based on our previous studies (13–15) were designed for human studies under the exposure to 60 GHz MMW (Figure 1). However, we found that a sufficient space between the wave source and the exposure target was needed to take the two-dimensional images. Therefore, main requirements to develop our MMW exposure setup were as follows:

1) To realize highly localized MMW exposure spot on the human skin in a predetermined area with desired power densities and frequencies
2) To keep enough space between the human body and the exposure antenna for measuring temperature rise and biological reactions simultaneously in real time during the MMW exposure.

Based upon these requirements and our technical experiences, we redesigned specifications for the exposure setup construction as summarized in Table 1.

**Table 1:** Specifications for developed exposure setup.

Exposure frequency [GHz]	60 +/– 0.5
Focal length [mm]	300
Diameter of focused exposure area (@-3 dB) [mm]	20
Handling averaged power density on the exposure area [W/m^2^]	0–1940

### Evaluation of the Exposure Setup Using FDTD Method

A focused beam exposure system using a dielectric lens has been investigated (14–18) as MMW exposure equipment. In this study, a lens antenna with a focal length of 300 mm and a 3 dB beamwidth of 20 mm at 60 GHz were developed. We firstly estimated the maximum output power of MMW amplifier that was required to detect some biological effects of the exposure. Although it was observed in the exposure experiment with 28 GHz MMW, the local MMW exposure at 1,800 W/m^2^ of IPD elevated about 7°C of skin temperature in the human forearm (14). Therefore, at least this IPD value was likely to be necessary to evoke changes in several physiological parameters, because the warm detection temperature was reported to be 32.2–40.7°C in the human skins of several body regions, which means the temperature rise was 0.2–8.7°C from the starting temperature of 32°C (12). Based on these findings, the maximum output power of MMW amplifier was estimated as 4 W or more in the case of using the single lens antenna of 60 GHz.

**Figure 2:**
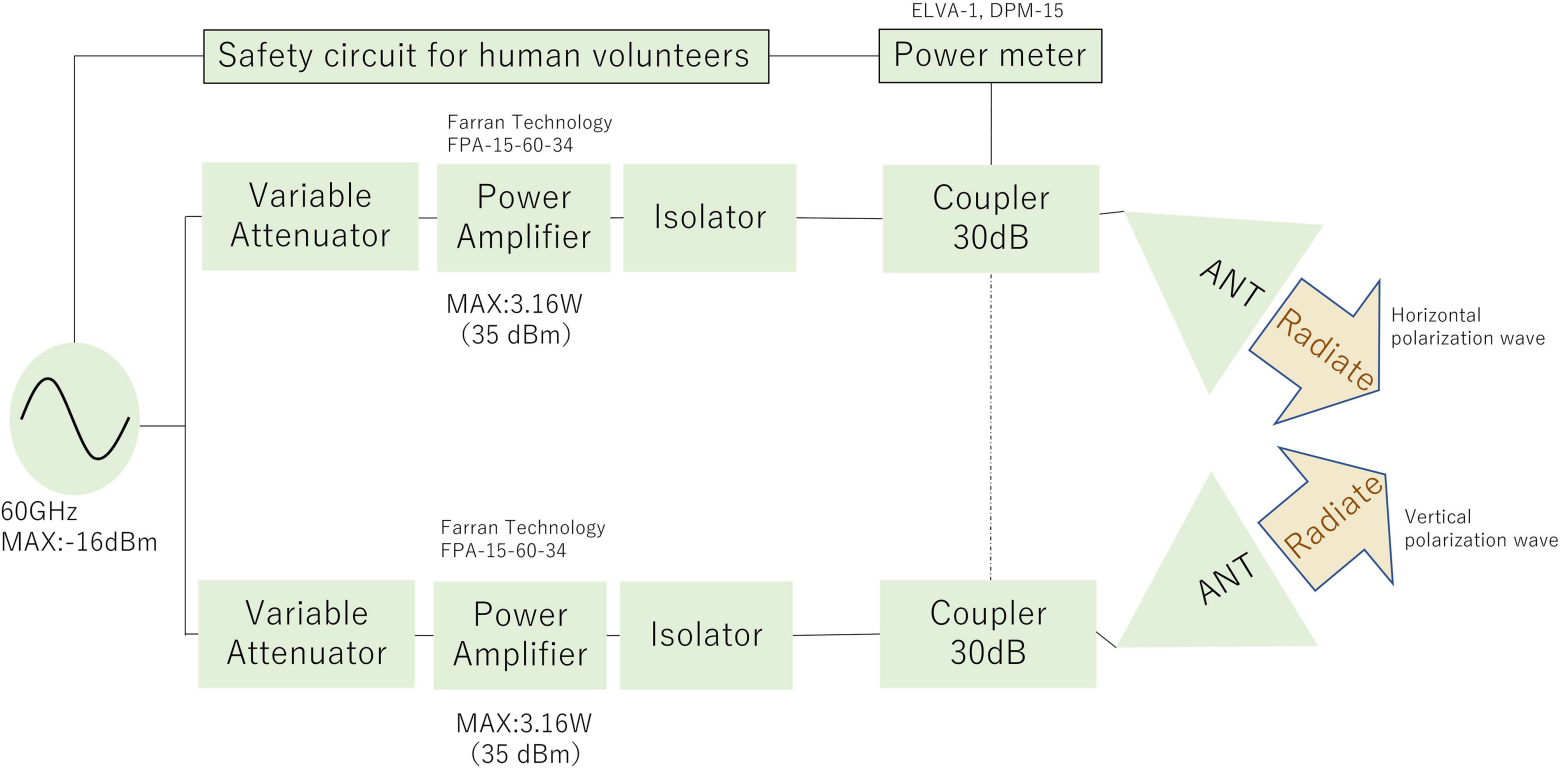
Designed block diagram of the proposed exposure setup using spatial synthetic technique.

However, it was difficult to obtain a current commercially available stand-alone amplifier with such a high output power in the 60 GHz band. Even if a traveling wave tube amplifier or impact ionization avalanche transit-time diode is used, the dimensions of the exposure device will be huge. In addition, these devices are incapable of exposure using 5G/beyond 5G modulated signal and cause loud noise and low power-handling problems. Consequently, these devices seemed to be unsuitable for experimental studies in human volunteers. Therefore, we proposed the application of a spatial synthesis beam technique that could achieve a total averaged output power of 5 W or more. Figure 2 shows the designed block diagram of the exposure setup. The 60 GHz MMW signal from the signal generator was divided into two waveguides. Each signal was amplified, and the focused beams were radiated from the antennas with the same amplitude and phase.

**Figure 3:**
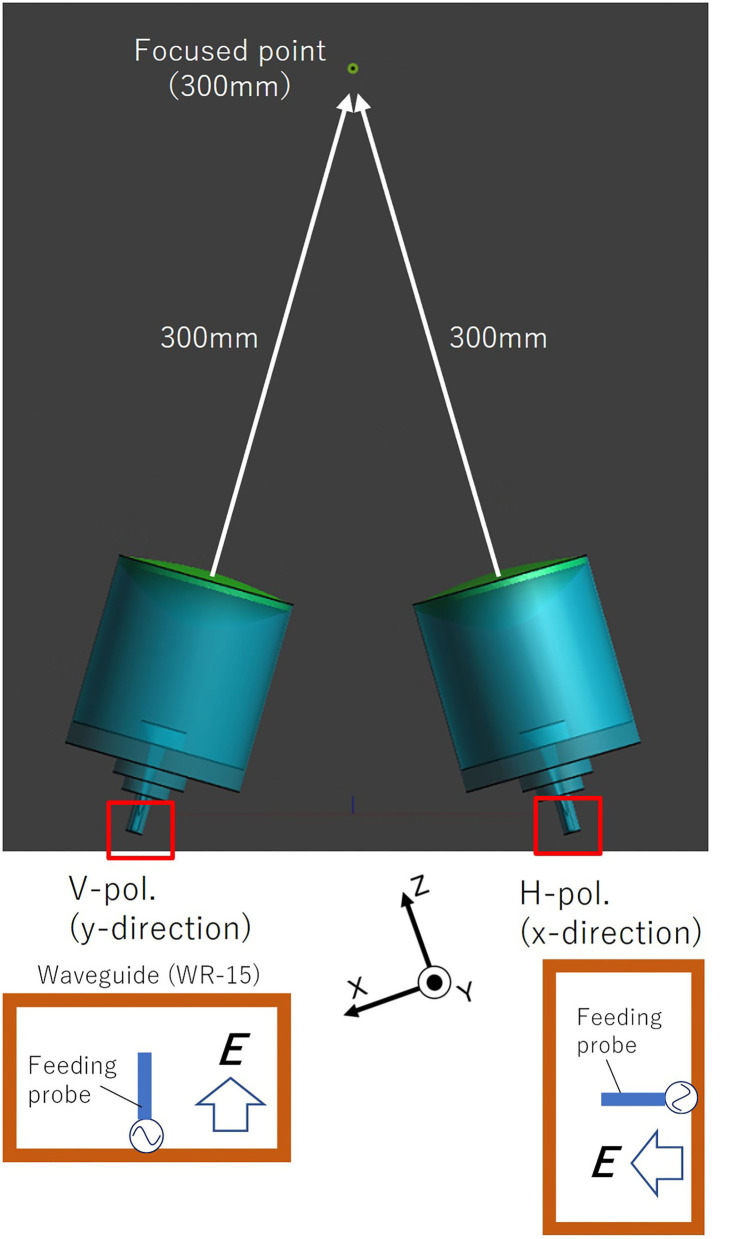
Orthogonal polarized feeding for spatial synthetic beam.

**Figure 4:**
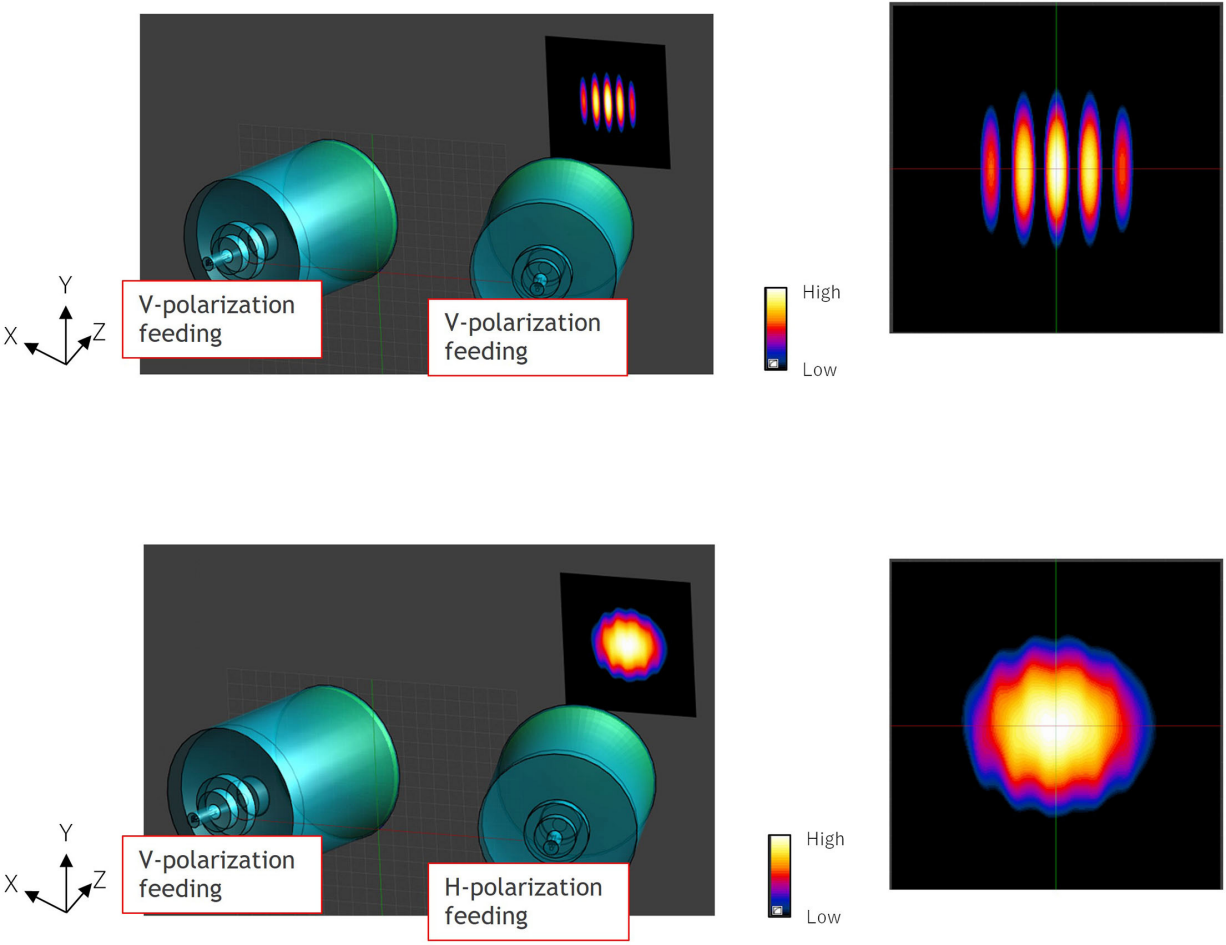
Electric field distributions on a flat planer skin phantom exposed to spatial synthetic beams at 60 GHz. The values were normalized by the maximum value. **(A)** Copolarized feed. **(B)** Orthogonally polarized feed.

However, interference fringes were generated in the exposure area when the beam radiations from two directions were combined on the spot, even if their amplitude and phase of the antenna input signal are perfectly coincident. To eliminate the interference fringes, we also proposed to apply to orthogonalize the polarization of the two antennas, as shown in Figure 3. We estimated electromagnetic fields on the exposure area irradiated by focused beams, using commercial finite-difference time-domain (FDTD) software (Sim4Life V 5.2.2.1924, Schmid & Partners Engineering AG, Zürich, Switzerland). Based upon the simulation results, we designed that the angle between the two lens antennas for spatial synthesis was set at 36° in considering the size of the lens antenna element and the focal length of 300 mm. Resultantly, the incident angle of each beam radiated from the antenna became not perpendicular (±18°) to the surface of exposure plane. As well known, transmission and reflection coefficients are varied depending upon the incident angle and polarization characteristics (19). We also estimated the exposure characteristics due to the polarization angle of diagonal incidence irradiation using the FDTD simulation. It was confirmed that the effect due to the different incident angles of ±18° relative to normal was approximately < 0.1 dB in absorbed power density (20, 21), and it was negligible. Estimated electric field distributions on a numerical flat plate that dielectric parameter was the same as dry skin exposed to spatial synthetic beams at 60 GHz. Figures 4A,B show the results for applying the copolarized feed and the orthogonally polarized feed, respectively. From the figures, interference fringes in the exposure area were highly suppressed by applying orthogonalizing polarized feed. The size of the focused circle on the exposure plane was 20 mm in diameter in half-power width. We confirmed that the reason why the interference fringes were not completely suppressed was due to the crosspolarization property of the lens antenna and the reflection on the phantom surface.

## ARM-Shaped Silicone Phantom for Basic Performance Measurement

A solid phantom material composed of carbon nanotubes, silicone rubber, and carbon black (CB) was developed to evaluate the basic performance of the developed 60 GHz band exposure setup. By using the novel phantom, it can be safely confirmed that the exposure setup meets the required specifications before human studies. The carbon nanotubes took the form of cylindrical carbon molecules, with at least one end-capped with a hemisphere of the structure. The diameter of a carbon nanotube was the order of a few nanometers, and it was up to several micrometers in length. Carbon black was a form of amorphous carbon that had an extremely high surface area to volume ratio. By using these carbon materials mixed within silicone rubber, an arm-shaped human skin phantom was suitable for 60 GHz band. This phantom material's complex permittivity was controlled by changing the composition ratio of carbon nanotubes and carbon black within the silicone base. The penetration depth of the MMW in biological tissue was minimal, and it became approximately 0.4 mm at 60 GHz band. Therefore, the target value of dielectric properties of the phantom was decided as the averaged value of skin tissues, and those are summarized in Table 2.

**Table 2:** Dielectric properties of skin tissues (22).

	**Relative permittivity**	**Loss tangent**
Skin dry	7.9	1.4
Skin wet	10.2	1.2
Developed material	12	1.2

## Experimental Measurements and Discussions

### Incident Power Density Measured at the Target Area

**Figure 5:**
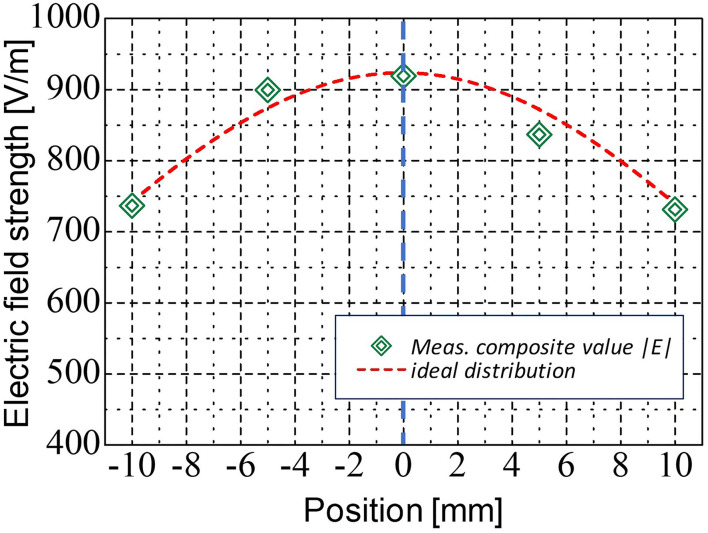
Measured composite electric field distribution of orthogonal polarization lens antennas at 1 W of total antenna input power.

To estimate the exposure characteristics when the orthogonal antennas were used, we measured electric field distribution on the exposed area using a V-band open-ended waveguide probe antenna (SAGE Millimeter, Inc SAP-15-R2). Based upon the conversion formula between the probe antenna receiving power and electric field intensity, we evaluated the electric field distribution of each orthogonal polarization component from each antenna with total input power of 1 W, and the composite distribution was obtained. When the interference fringes are generated in the exposure area, the fluctuation of receiving power along with the measured line should be clearly observed. As shown in Figure 5, it was found that the exposure intensity distribution of the spatial synthetic beam was almost identical to that of single-focused beam irradiation. Thus, the suppression of the interference fringes by applying orthogonalizing polarized feed was confirmed. In addition, we found that the developed exposure systems could achieve the free-space power density of 970 W/m^2^ when the total antenna input power was set to be 2 W.

### Verification of the Simulated Results Using the Forearm Phantom

**Figure 6:**
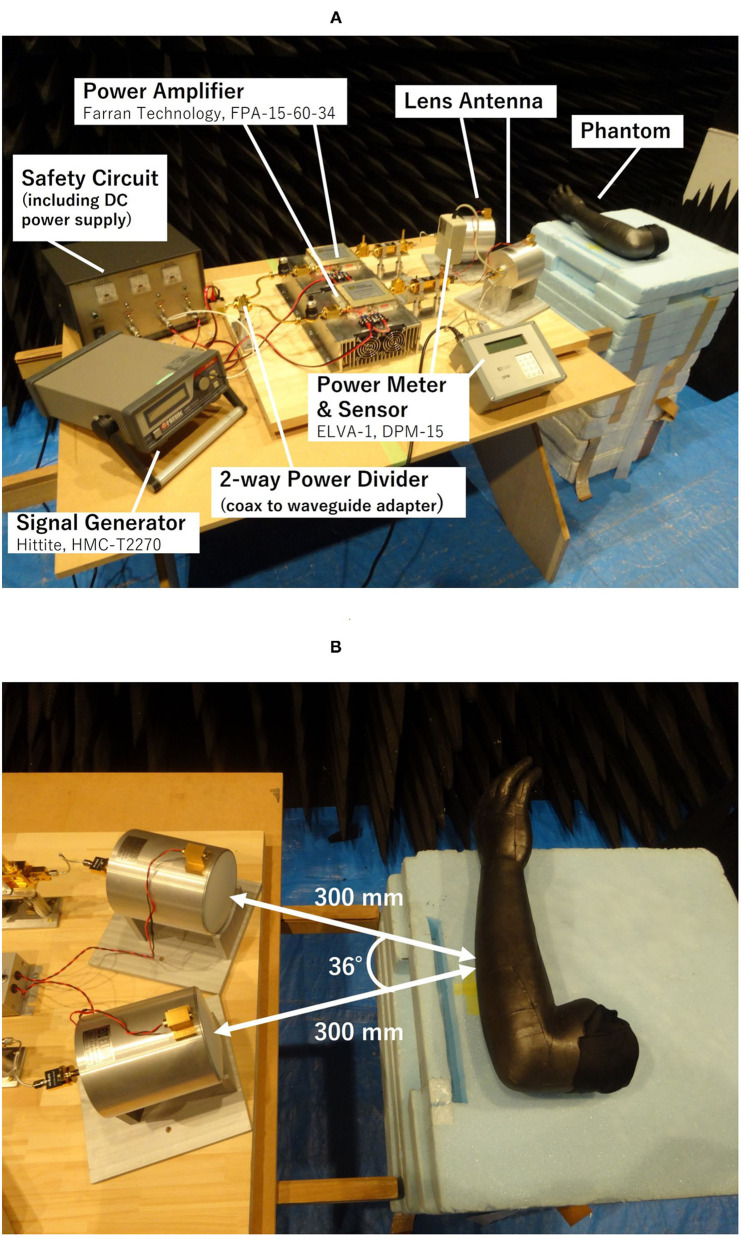
60 GHz spatial synthetic exposure setup. **(A)** Overview of the setup. **(B)** The lens antenna and arm-shaped phantom.

Based upon the designed block diagram and simulation results, we decided the set position and angle of the lens antennas and constructed the spatial synthetic exposure setup. Figures 6A,B show an overview of the developed setup in the anechoic chamber and an enlargement of the lens antennas and the arm-shaped phantom, respectively. Most of the MMW components were connected by rectangular waveguides of WR-15. The two lens antennas were connected to the lines with low-loss semirigid coaxial lines to control the feeding polarization and the angle between the two lens antennas.

**Figure 7:**
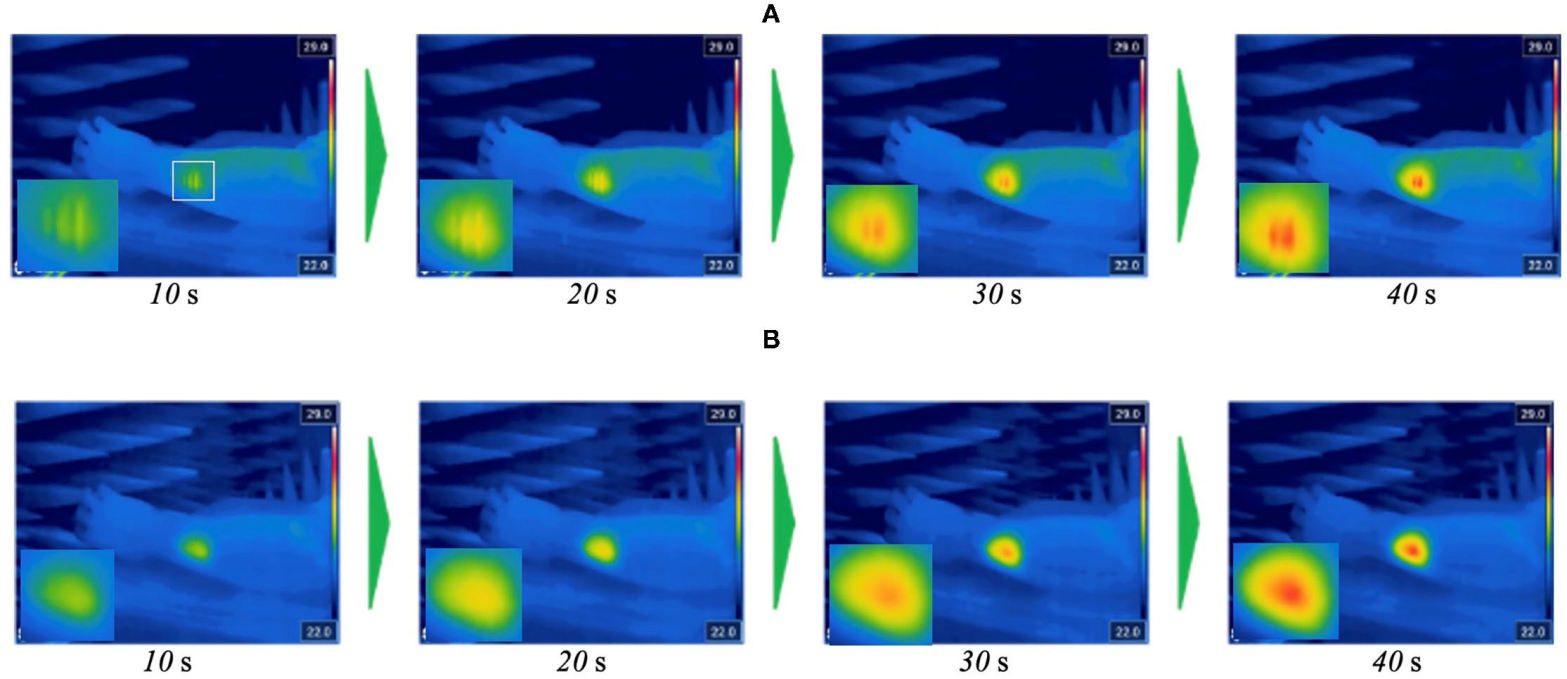
Temperature distribution on arm-shaped phantom during the exposure to 60 GHz MMW at 2 W of total antenna input power. **(A)** Orthogonally polarized feed. **(B)** Copolarized feed.

Figure 7 shows surface temperature distributions of the arm-shaped phantom observed by thermal imaging camera (FLIR T530, FLIR Systems, Inc. USA) during exposure to 60 GHz-MMW at 2 W of total antenna input power. The characteristics of temperature distribution after the beginning of the exposure are shown every 10 s until 40 s. Interference fringes were clearly observed on the phantom surface when 60 GHz MMW radiation from copolarized feeding antennas was combined. In contrast, orthogonalizing the polarization of the two antennas eliminated these interference fringes. From the figure, it was confirmed that the exposure focused on a circular area of about 20 mm in diameter was realized.

**Figure 8:**
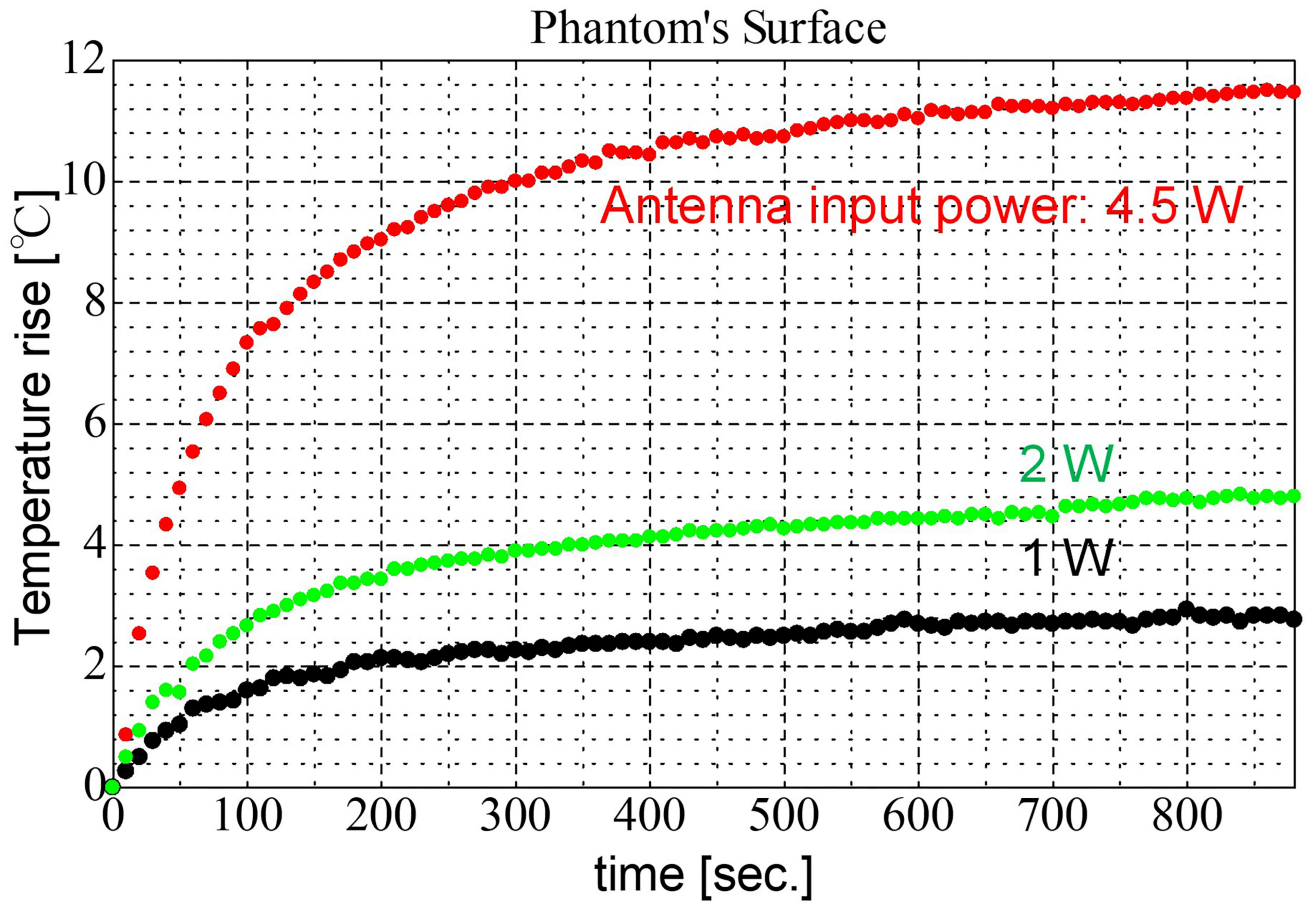
Characteristics of temperature elevation during the exposure to 60 GHz MMW with orthogonal polarized feeding.

The time- and dose-dependent characteristics of temperature rise were also evaluated using the phantom forearm under the measurement conditions shown in Table 3. These conditions except the room temperature were set to be similar to those in our future studies using human volunteers. Orthogonally polarized feed was adopted. The temperature of the phantom surface was measured when the antenna input power (total of two antennas) was at 1.0 W, 2.0 W, and 4.5 W. Figure 8 shows a comparison of the characteristics of the temperature rise at target surface for each antenna input power. It is confirmed that the developed exposure setup provided the ideal local exposure on the target area. The surface temperatures elevated time-dependently similar to those we observed in the previous study using 28 GHz MMW. In addition, the maximum temperatures depended on the antenna input power, suggesting the differences in the IPD values, were reflected in the temperature elevation. However, the further experiments with several exposure intensities were needed to verify the linear relationship between the exposure intensity and temperature elevation.

**Table 3:** Experimental conditions for measuring temperature elevations in phantom.

Room temperature	20°C
Exposure frequency	60.0 GHz
Duration	0~900 sec.
Total input power for two antennas	1, 2, 4.5 W

### Confirmation of the Local MMW Exposure in Human Forearm Skin

It was important to confirm whether the similar characteristics of local MMW exposure observed in the simulation and phantom experiment were actually obtained in human skin. Then, we conducted human volunteer experiments using the developed exposure setup. Six adult volunteers (five female volunteers and one male volunteer) were called and explained our studies in detail to obtain informed consent. The experiment was performed in a room kept at 26.5 ± 0.5°C of temperature and 45 ± 5% of relative humidity. Subjects sat in the chair and their right forearms were placed on the desk. The dorsal forearm skin positioned 300 mm in front of the lens antenna was exposed to 60 GHz MMW at 2 W of total antenna input power for 6 min. Changes in skin temperature were measured through the experiments using thermography. This study was approved by the Ethics Committee of Kurume University (approved number 17192).

**Figure 9:**
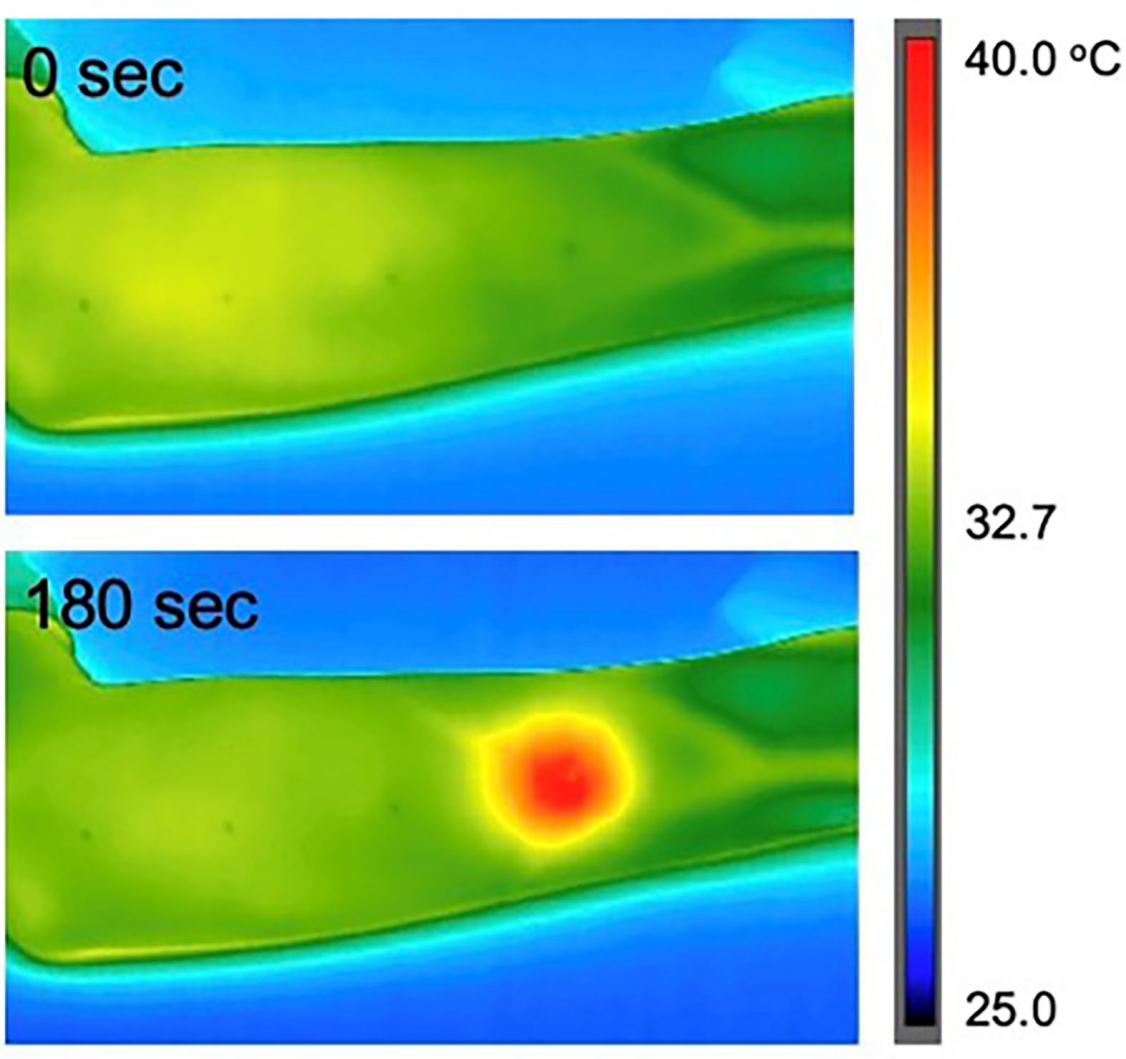
Temperature elevation in the human forearm skin during the exposure to 60 GHz MMW at 2 W of total antenna input power.

Figure 9 shows representative images of thermography around the target area during the exposure. The temperature elevation was observed and found it localized at the target area. In addition, no striped distribution of temperature was confirmed. Although the thermoregulation or thermal diffusion in the skin should be taken into consideration for the temperature distribution, these findings were very similar to those of the phantom experiment. Furthermore, the average temperature of the target area exposed at 2 W of total antenna input power increased from 32.3 ± 0.9°C to 37.7 ± 1.0°C. This range of temperature change seemed to be enough for warm detection reported in the skins such as thenar and dorsal hand (32.3–35.3°C) (12). Therefore, it suggests that the local MMW exposure that we expected for human studies is realized in the human skin.

## Conclusion

We developed a novel spatial synthetic exposure setup to explore the biological effects of local exposure to 60 GHz-MMW on human body. The setup using double lens antennas allowed to generate the highly localized exposure area on the human skin and provided enough space for measurements of the physiological parameters. In addition, even at 300 mm of the focal length between the target area and the antenna, the sufficient IPDs (0 to 1,940 W/m^2^) to detect thermal threshold were obtained. This is the first report, to the best of the authors' knowledge, about the developments of the MMW spatial synthetic exposure setup using orthogonally polarized feeding structure to eliminate interference fringes in the exposure area. On the other hand, purposely using the interference fringes may also be capable of achieving more localized exposure with high intensity, namely multiple exposures using a synthetic electric field instead of composite power. The developed exposure setup will be useful for the future human studies. For example, the effects of 5G-MMW exposure on skin thermoregulation under various exposure conditions such as different IPDs or signal modulation will be considered.

## Data Availability Statement

The original contributions presented in the study are included in the article/supplementary material, further inquiries can be directed to the corresponding author.

## Ethics Statement

The studies involving human participants were reviewed and approved by Ethics Committee of Kurume University. The patients/participants provided their written informed consent to participate in this study.

## Author Contributions

TH designed all the experiments and simulations and conceived the manuscript. RO performed the experiments and wrote the draft of the manuscript. HM and TI reviewed the manuscript. All authors have read and agreed to the published version of the manuscript.

## Funding

This study was supported by Ministry of Internal Affairs and Communications in Japan. Grant number JPMI10001.

## Conflict of Interest

The authors declare that the research was conducted in the absence of any commercial or financial relationships that could be construed as a potential conflict of interest.

## Publisher's Note

All claims expressed in this article are solely those of the authors and do not necessarily represent those of their affiliated organizations, or those of the publisher, the editors and the reviewers. Any product that may be evaluated in this article, or claim that may be made by its manufacturer, is not guaranteed or endorsed by the publisher.
